# Surveillance for respiratory viruses in freshwater bodies visited by migratory birds, the Philippines

**DOI:** 10.5365/wpsar.2024.15.3.1123

**Published:** 2024-08-12

**Authors:** Romeo S Gundran, Dan Drexel Dela Cruz, Milagros R Mananggit, Joely T Ongtangco, Xandre D Baccay, Ronnie D Domingo, Mary Elizabeth G Miranda, Emily Bailey, Samantha Gabrielle Cody, Laura A Pulscher, Emily R Robie, Gregory C Gray

**Affiliations:** a Central Luzon State University, Science City of Muñoz, Nueva Ecija, Philippines.; b Department of Agriculture, Regional Field Office III, Pampanga, Philippines.; c Bureau of Animal Industry, Quezon City, Philippines.; d Field Epidemiology Training Program Alumni Foundation, Manila, Philippines.; e Department of Public Health, Campbell University, Buies Creek, North Carolina, United States of America.; f Division of Infectious Diseases, University of Texas Medical Branch, Galveston, Texas, United States of America.; g Duke Global Health Institute, Duke University, Durham, North Carolina, United States of America.; h Institute for Human Infections and Immunity, University of Texas Medical Branch, Galveston, Texas, United States of America.; i Department of Microbiology and Immunology, University of Texas Medical Branch, Galveston, Texas, United States of America.; j Department of Global Health, University of Texas Medical Branch, Galveston, Texas, United States of America.

Migratory birds are known to spread influenza viruses, and the Philippines is a common stopover for several species that fly long distances. (*1*) Influenza A viruses are a particular global health concern because of their zoonotic potential, impacting not only humans but also animals and wildlife. In 2005, thousands of migratory birds died from avian influenza at a major migratory stopover point in western China. (*2*) The Philippines is an important aggregation and breeding site for migratory birds that are distributed across central Asia. In September, they migrate southwards from China to Myanmar and over the Himalayas to India, returning to China around April. Through this migration, avian influenza viruses can spill over to new bird species, including domestic poultry, across countries and potentially to humans. (*2*)

In the Philippines, ducks are a common domestic avian species and an important source of meat and eggs for the communities that raise them. However, since outbreaks of highly pathogenic avian influenza (HPAI) occurred in the Philippines in 2017, duck production in both commercial and backyard settings has decreased, resulting in economic strain and food insecurity. (*3*, *4*) Government authorities suspected the HPAI outbreaks were linked to bird migration. (*3*) However, because of the temporary nature of migratory birds’ residence across their flyways, trapping and sampling them is difficult, prompting researchers to seek alternative ways to detect HPAI viruses. Sampling from water bodies frequented by migratory bird populations has been proposed as an efficient and effective alternative means of detecting the viruses. (*5*) In the Philippines, water bodies such as riverbanks, creeks, marshlands, irrigation canals, rice fields and bird sanctuaries are known to harbour migratory bird populations and are likewise used as grazing sites for domestic ducks.

Sampling water to study the prevalence of avian influenza viruses began in the late 1970s and has been periodically used since then. (*6*) In one 2014 study in China, investigators were able to culture H5N1 and H9N2 avian viruses from natural water bodies for up to 45 days after migratory birds had stopped there. (*6*) In 2020, water sampling became even more widely used due to the COVID-19 pandemic, (*7*) during which the discovery of severe acute respiratory syndrome coronavirus 2 (SARS-CoV-2) in water bodies was reported, and since then wastewater-based epidemiology has been applied as an early surveillance tool. (*8*) In this paper, we employed water sampling as a method for assessing avian-to-avian transmission and the potential for spillover of HPAI virus strains from migrating birds to domestic poultry in the Philippines.

## Methods

### Sample collection

**Fig. 1:**
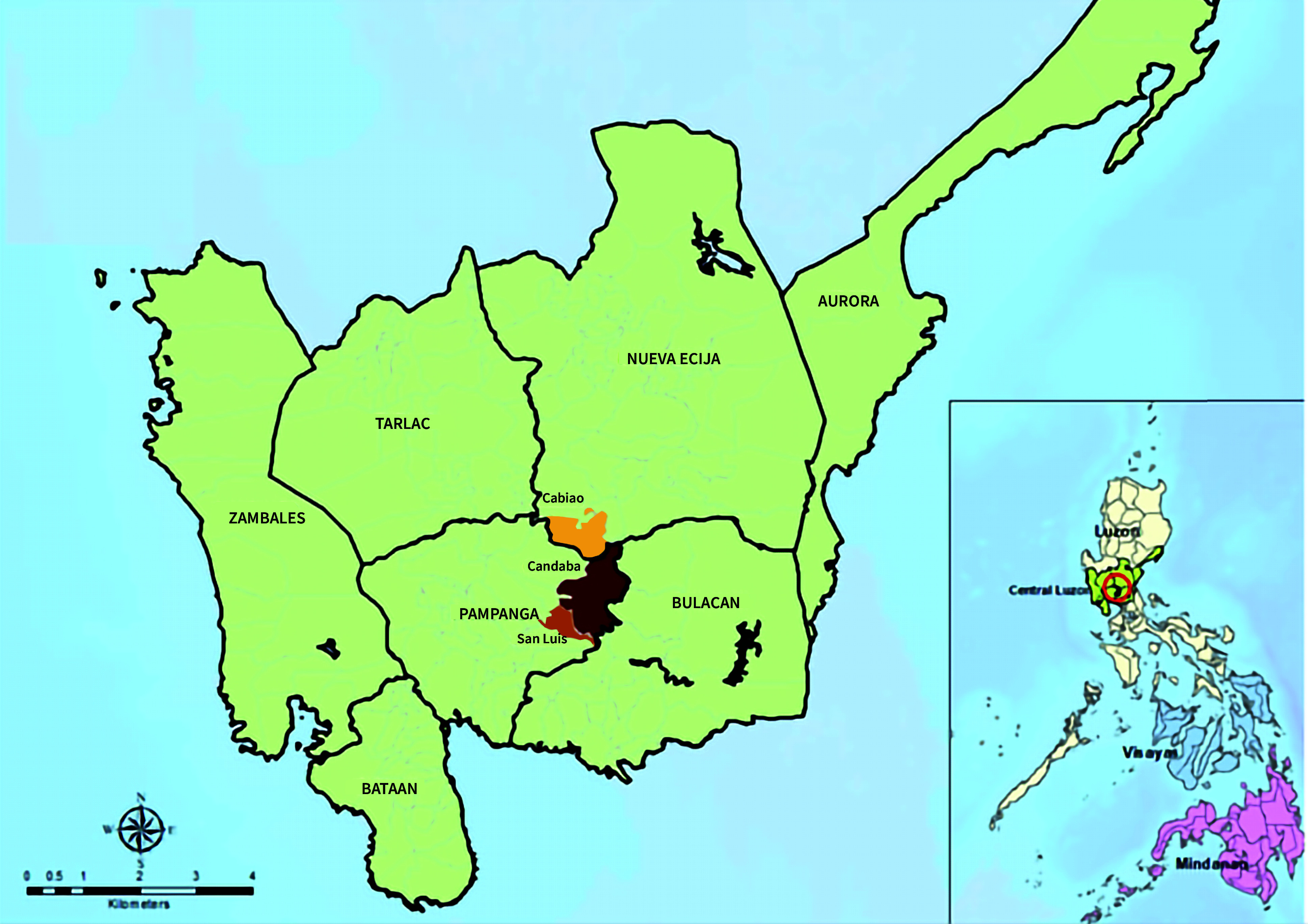
Map of Central Luzon Region, the Philippines, showing the three municipalities where water sampling was conducted (Cabiao, Nueva Ecija province; Candaba and San Luis, Pampanga province), 2019–2020

Three areas visited by migratory birds in the Central Luzon Region of the Philippines were selected for sampling: Cabiao in Nueva Ecija province, San Luis in Pampanga province and Candaba in Pampanga province (**Fig. 1**). From October 2019 to August 2020, samples of environmental water were collected on five different occasions from six bodies of water, such as bird sanctuaries, riverbanks, creeks, marshlands, irrigation canals and rice fields, where migratory birds and ducks are typically seen during the migration season and where commercial and backyard ducks also commonly graze. In the Philippines, the southward migration of birds typically peaks between September and November, while the northward migration peaks between February and April. Google Maps was used to create a sampling grid to determine the sampling points. Samples were then randomly collected from within the identified sampling points. From each identified sampling spot for each body of water, 10 water samples of 50 millilitres (mL) each were taken at least 5 metres apart. Next, for each group of 10 samples, the samples were combined and then divided into two pools for each body of water from each municipality.

[**insert** Figure 1]

During sample collection, the following information was obtained: date and time the sample was collected, water temperature, water pH, type of vegetation in the area, and presence or absence of migratory birds. Fifty mL of viral transport medium was added to each 50-mL sample, and these 100-mL field samples were transported at ambient temperature for testing at the Regional Avian Influenza Diagnostic Laboratory in Pampanga, Philippines.

### Laboratory testing

Samples were processed and molecularly screened for adenovirus, enterovirus, coronavirus, influenza A virus and influenza C virus based on previously published methods. (*9*) The 100-mL water samples were vortexed and a 200-µL sample from each was pooled by location and date into one sample before nucleic acid extraction. RNA and DNA were extracted from the samples using the QIAamp MinElute Virus Spin Kit (Qiagen, Germantown, MD, USA). Samples were then screened by quantitative reverse transcription–polymerase chain reaction (qRT–PCR) for the influenza A virus matrix gene using primers and probes. Samples were also examined by qRT–PCR or qPCR assays for influenza C virus, adenovirus, seasonal coronaviruses and enteroviruses. (*9*) Samples positive for influenza A underwent haemagglutinin subtyping at the Research Institute for Tropical Medicine in Manila, Philippines.

## Results

From October 2019 to August 2020, a total of 180 samples were collected (60 samples per municipality). This resulted in 36 pooled samples, 12 from each municipality.

The samples were collected in waters of different pH (range: 7.0–9.3), varying temperatures (range: 26–38 °C) and different vegetation types (**Table 1**). Various wild birds were observed in the waters including brown and white sparrows (*Passer montanus*), buff-banded rails (*Gallirallus philippensis*), mallard ducks (*Anas platyrhynchos*), storks (*Anastomus oscitans*) and wild ducks (*Anas luzonica*) (**Table 1**).

**Table 1:** Summary of water samples collected from three municipalities in the Philippines, 2019–2020

Municipality, province, type of water body	pH	Temperature (°C)	Vegetation type present^d^	Migratory birds present^e^	Collection date	Collection time
**Cabiao, Nueva Ecija**						
**Riverbank**	**8.7**	**29**	**Camachile, tall grass, water hyacinth, water lilies**	**Brown sparrow**	**8 November 2019**	**14:16**
**Creek^a^**	**7.7**	**26**	**Water hyacinth, water lilies**	**Brown sparrow, stork**	**29 October 2019**	**15:06**
**Marshland**	**8.2**	**37**	**Camachile, para grass, vines, water spinach, wild tamarind**	**Stork**	**8 November 2019**	**13:28**
**Irrigation canal**	**8.4**	**38**	**Para grass**	**Brown sparrow, stork**	**8 November 2019**	**12:58**
**Rice field**	**–^c^**	**–^c^**	**Para grass**	**Stork**	**28 August 2020**	**10:36**
**Bird sanctuary**	**8.4**	**32**	**Wild spinach**	**Brown sparrow, buff-banded rail, stork**	**8 November 2019**	**10:45**
**Candaba, Pampanga**						
**Riverbank^b^**	**8.5**	**31**	**Bamboo, banana, para grass, tall grass**	**Brown sparrow**	**7 November 2019**	**13:01**
**Creek**	**7.0**	**33**	**Tall grass, water spinach**	**Brown and white sparrows, stork**	**31 October 2019**	**14:05**
**Marshland**	**7.7**	**32**	**Water hyacinth, water lilies, water spinach**	**Stork**	**31 October 2019**	**13:35**
**Irrigation canal**	**7.8**	**30**	**Wild tamarind**	**Stork**	**31 October 2019**	**13:01**
**Rice field**	**8.6**	**32**	**Para grass, water spinach**	**Brown sparrow, stork**	**8 November 2019**	**13:25**
**Bird sanctuary**	**7.3**	**31**	**Lotus, eucalyptus tree, water hyacinth, water lilies, wild spinach, wild tamarind**	**Brown sparrow, stork, wild duck**	**31 October 2019**	**13:43**
**San Luis, Pampanga**						
**Riverbank**	**8.3**	**27**	**Tall grass, water hyacinth, water lilies**	**Brown sparrow**	**7 November 2019**	**12:02**
**Creek**	**8.5**	**27**	**Carabao grass, tall grass, water hyacinth water lilies**	**Brown sparrow, mallard duck, stock**	**7 November 2019**	**12:15**
**Marshland**	**9.3**	**31**	**Acacia, water hyacinth, water lilies**	**Stork**	**7 November 2019**	**11:15**
**Irrigation canal**	**8.0**	**26**	**Tall grass**	**Brown sparrow**	**7 November 2019**	**10:40**
**Rice field^b^**	**8.1**	**27**	**Rice grass**	**Stork**	**31 October 2019**	**11:30**
**Bird sanctuary**	**8.2**	**27**	**Water spinach, water hyacinth, water lilies**	**Brown sparrow, stork, other birds**	**28 November 2019**	**10:45**

^a^ Positive for influenza A.

^b^ Positive for enterovirus.

^c^ Measurements not recorded.

^d^ Acacia: *Acacia mangium*; bamboo: *Bambusa vulgaris*; banana: *Musa* spp.; camachile: *Pithecellobium dulce*; carabao grass: *Paspalum conjugatum*; eucalyptus tree: *Eucalyptus deglupta*; water hyacinth: *Eichhornia crassipes*; water lilies: *Nymphaea* spp.; lotus: *Nelumbo nucifera*; para grass: *Brachiaria mutica*; tall grass: *Saccharum spontaneum*; vines: *Ipomoea batatas*; water spinach: *Ipomoea aquatica*; wild spinach: *Amaranthus viridis*; wild tamarind: *Leucaena leucocephala*.

^e^ Brown or white sparrow: *Passer montanus*; buff-banded rail: *Gallirallus philippensis*; mallard duck: *Anas platyrhynchos*; stork: *Anastomus oscitans*; wild duck: *Anas luzonica*.

Of the pooled samples, one pool (2.78%) from a creek in Cabiao was positive for influenza A virus, and two pools (5.55%) were positive for enterovirus: one from a riverbank in Candaba and one from a rice field in San Luis (**Table 1**). The detection of influenza A virus was consistent with the H9 virus identified by haemagglutinin subtyping. The molecular assays for influenza C virus, adenovirus and seasonal coronaviruses on the pooled specimens were all negative.

## Discussion

The Philippines serves as a crucial stopover and wintering site for many migratory bird species. (*10*-*15*) As a common resting place for migratory birds, the country is at risk for an avian influenza spillover to domestic poultry, and potentially humans, due to the mixing of many wild bird species from across Australia, Asia and Oceania. Environmental sampling of various water bodies frequented by both domestic and wild birds yielded one pooled sample positive for influenza A virus from an area that, at the time of sampling, was inhabited by brown sparrows and storks. Two pooled samples were also positive for enterovirus. The first was collected in an area where brown sparrows were present and the second where storks were present. Notably, these same species were also seen frequenting sites where no virus was identified in the samples. Both kinds of birds are migratory and not permanent residents of the Philippines, confirming that the selected sites do serve as resting spots for such migratory birds, which may visit these waters twice annually. Influenza A virus may be transmitted from the carrier to the environment through faeces, which could then be spread to new avian species through contaminated water bodies. Similarly, enteroviruses can also be spread through environmental water sources. (*16*)

This pilot study was limited in that relatively few (*n* = 180) specimens were collected in a relatively narrow geographical region on five occasions during a 10-month period. Additionally, relatively few viruses were detected in these samples, and detailed characterization of the influenza A and enteroviruses that were detected was not performed. Our study was further limited in that the adenovirus assay used in this study was developed to detect human adenoviruses and may have missed non-human adenovirus strains. Hence, the sampling was not comprehensive, and important viruses may not have been detected. Regardless, our study found evidence that freshwater bodies can harbour influenza A virus. The virus can be shed by migratory birds through their faeces, and such contaminated water bodies may transmit the virus to livestock and possibly to humans. These results highlight the significant risk posed by the practice of allowing domestic ducks to forage near freshwater bodies visited by migratory birds. Ducks, once infected, can potentially spread the virus to other birds and sometimes to humans. This study may serve as an example of an alternative strategy for surveillance of avian influenza viruses among migratory birds. Our hope is that water surveillance might help to mitigate HPAI infections among poultry, such as the epizootics that occurred in the Philippines during 2017 and January 2022. (*17*)
